# BitTorious: global controlled genomics data publication, research and archiving via BitTorrent extensions

**DOI:** 10.1186/s12859-014-0424-9

**Published:** 2014-12-21

**Authors:** Preston V Lee, Valentin Dinu

**Affiliations:** Department of Biomedical Informatics, Arizona State University, 13212 East Shea Boulevard, Scottsdale, AZ 85259 USA

**Keywords:** Networking, Data transfer, Bioinformatics, Big data, Software, Open source, Bittorrent

## Abstract

**Background:**

Centralized silos of genomic data are architecturally easier to *initially* design, develop and deploy than distributed models. However, as interoperability pains in EHR/EMR, HIE and other collaboration-centric life sciences domains have taught us, the core challenge of networking genomics systems is not in the construction of individual silos, but the *interoperability* of those deployments in a manner embracing the heterogeneous needs, terms and infrastructure of collaborating parties. This article demonstrates the adaptation of BitTorrent to *private* collaboration networks in an authenticated, authorized and encrypted manner while retaining the same characteristics of standard BitTorrent.

**Results:**

The BitTorious portal was sucessfully used to manage many concurrent domestic Bittorrent clients across the United States: exchanging genomics data payloads in excess of 500GiB using the uTorrent client software on Linux, OSX and Windows platforms. Individual nodes were sporadically interrupted to verify the resilience of the system to outages of a single client node as well as recovery of nodes resuming operation on intermittent Internet connections.

**Conclusions:**

The authorization-based extension of Bittorrent and accompanying BitTorious reference tracker and user management web portal provide a free, standards-based, general purpose and extensible data distribution system for large ‘omics collaborations.

## Background

Etymology of the term “warehouse”, by definition, evokes imagery of a single, huge, meticulously organized building filled with densely stacked shelves, pallets and boxes. In informatics, however, we often abstract physical implementation behind logical representation. This paper leverages the physically distributed properties of BitTorrent to create a massive logical data warehouse.

Taking an architectural lesson from data transfer solutions of decades past, spanning the emergence of decentralized distribution (such as BitTorrent) over the Internet, multicast UDP, even interplanetary networking -- it may be that the *essential complexity* [1] of globalized “big data” fields is of sufficient magnitude that congruity may not emerge organically. Given additional strong economic and practical interest in bioinformatic cloud computing [2], bioinformaticians sorely need sophisticated data transfer solutions to shuffle data between wet lab and IaaS environments in a controlled manner. We present BitTorious as a free and open tool for private data syndication in translational genomics and other data intensive fields.

**Figure 1:**
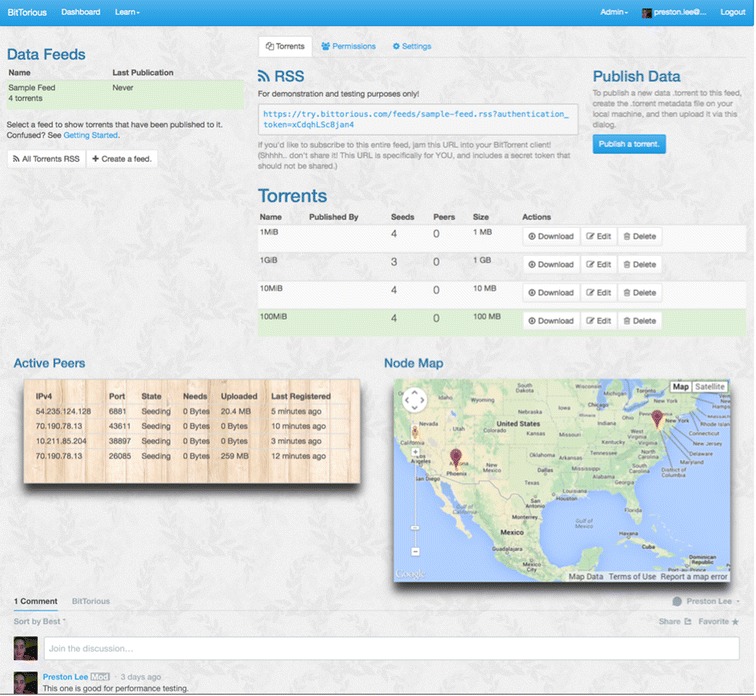
**BitTorious dashboard for a sample feed.** The BitTorious dashboard, allowing for access management, peer tracking, discussion, and data syndication.

A primary architectural benefit of distributed data distribution protocols such as BitTorrent, upon which BitTorious builds, is to improve *overall* systems performance at scale, such that a new node may, given enough peers, use the maximum amount of available local bandwidth. Protocol variations such as transparent compression and use of UDP may improve effective throughput, but such design decisions cannot nullify hard bottlenecks in the network limitations of centralized silo distribution models. As previously demonstrated, existing BitTorrent technology exhibits better performance characteristics than traditional centralized transport methods such as FTP at large scales [3]. BitTorious unifies a standards-compatible BitTorrent tracker with a custom integrated management portal for investigators to implement controlled access restrictions on data feeds. This marriage of existing P2P “swarm” semantics with BitTorious’s secured web service GUI (Figure 1) and API both substantially reduces the resource needs of publishing parties and enables easier automated integration of workflow across collaborating institutions.

## Implementation

Foremost, any proposed standards for worldwide consideration should support high performance, high availably data transfer over existing Internet connections for computational locality and archiving. While no approach is universally best, in the case of genomics data we argue that modifying individual purpose-specific data warehouses to expose a common, generic transfer layer based on common interaction standards is most suitable.

### Core BitTorious concepts

BitTorious primarily addresses the needs of data transport across the Internet to/from a changing and heterogeneous set of peer nodes. Core ideas are a combination of existing popular concepts from the BitTorrent content distribution protocol, Really Simple Syndication (RSS) -- as used by content publishers and subscribers such as bloggers -- and modern web-based application concepts, as explained below.

### Torrent

A "torrent" refers to a set of files that is published as a cohesive package of data ready for distribution. The data can be a single file, directory of files, or any combination thereof, and is initially defined using client software that produces a .torrent *file*. The .torrent file contains basic metadata and checksum information for the data being published, but does *not* contain the data themselves.

### Publication

A user ready to publish data does so by registering the torrent with a data RSS “feed” via the BitTorious portal, and initiates the “seeding” process to other client subscribers via the local client software. It is expected, though not technically required, that the publisher set additional metadata on the torrent via the BitTorious portal so subscribers can find the content via future search features.

### Subscription

In the most basic case, an authorized user wanting a specific package uses the BitTorious portal to find a download link to the .torrent file that is then loaded into the users desktop download client. In the more advanced case, wherein a user wants a “subscription” to all torrents published to a given feed, the user enters the URL for the feed into their BitTorrent client instead. All current and future data published to the given feed will be automatically downloaded as it becomes available.

### Seeding

A Client that has a full or partial copy of a torrent will, by default, be available to distribute the data to other subscribers. Limits can be set on throughput usage, time of use, quality of service, hard download/upload caps, or be turned off completely for each individual client.

### Deployment components

From a deployment perspective, BitTorious has only two types of components: a centralized web application portal, and any number of clients, some of which many be semantically declared as “dedicated” to the task.

### Portal

The BitTorious portal is a cross-platform web application that supports the latest stable versions of mainstream web browsers. Specifically, support is included for Firefox (Windows, OSX, Linux), Chrome (Windows, OSX, Linux), Safari (Windows, OSX), and Internet Explorer (Windows).

Internally, the portal's web application stack utilizes the Ruby on Rails framework on top of a PostgreSQL RDBMS instance to store active peer connection data, system accounts, feeds, torrents and torrent metadata, but not the published data. Web front-end rendering and interactivity is provided by standard HTML5, CSS3 (Bootstrap) and JavaScript (with jQuery).

### Clients

A client is simply a machine running a standard BitTorrent client to seed and/or download content to/from feeds, respectively. After a publication machine completes the initial seeding process to a similar dedicated client in the cloud, for example, it can be taken offline without disruption of the network as a whole. As long as data on the aggregate of online client machines contains all pieces of a given torrent, the data can be downloaded to completion by any current or new client. This characteristic of collective data replication provides an extremely resilient, high-ability architecture.

Note that it is generally assumed that the throughput rates of the underlying file system greatly exceed the throughput available over Internet connections at most institutions. If this is the case -- which it almost certainly is -- higher capacity disks will likely prove better investments than higher throughput disks.

### Dedicated clients

The optional addition of "dedicated" clients adds another availability component to the high-availability nature of the client system. The notion of a "dedicated" client is purely semantic, and there is no technological difference versus a normal client, with the understanding that a dedicated client is expected to:

- Seed all torrents for the feed(s) to which it is dedicated.
- Have enough bandwidth and underlying storage space to appropriately accommodate the needs of the publisher and subscriber base. This requires estimation on behalf of the deployment team to provide enough underlying storage.

Every instance of the BitTorious portal manages and controls one “network”, though there are no restrictions on the number of networks that may be deployed. Most clients will only be able to use one network at a time for security reasons, though deployment of two physically adjacent clients on two logically different networks is not an issue.

### Management

**Figure 2:**
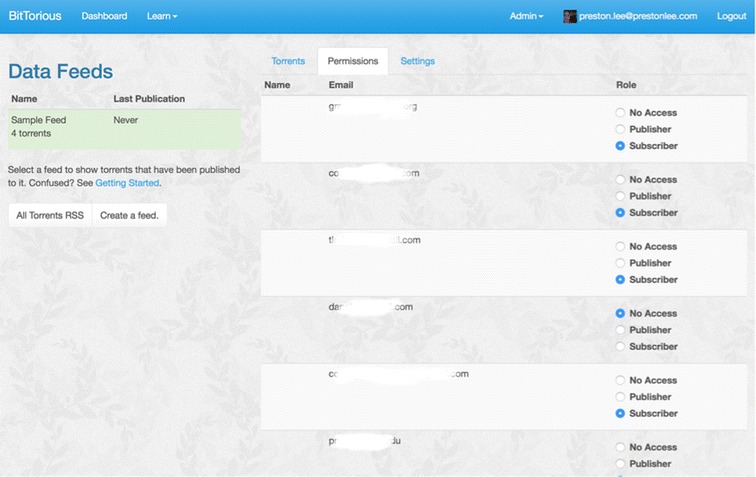
**Role-based access for user accounts.** Every data feed supports individualized role assignment for all users approved to access the network managed by the BitTorious portal.

Accounts are defined within the portal and are managed in a role-based manner. (Figure 2) Each role, below, is applied per user per feed.

- None – This is the default. When a user has not been granted access to a feed, they may neither view feed details nor join any torrents within it.
- Subscriber - The default role. May log into the portal, use download links, and join torrents.
- Publisher - All abilities as Subscribers, plus the ability to create, update and delete feeds.

Additionally, a global “Administrator” bit may be given to any user, which grants Publisher rights on all feeds, plus the ability to manage user accounts.

BitTorious groups torrents into access-controlled RSS feeds, allowing subscribers to automatically receive data based on the existing BEP-0036 draft specification. [4] The RSS URLs also include user-specific authorization tokens passed over encrypted SSL connections, allowing the tracker to securely authenticate every request. Since all peers are authenticated and authorized via combination of authorization token, SSL, and BEP-0036, the P2P protocol between peers remains unchanged.

### Security

Unlike standard BitTorrent, authentication and authorization checking are performed at three stages:

1. Web portal login.
2. Client data feed (RSS) update requests.
3. Client registration and keep-alive with the web portal.

BitTorious controls access to metadata and torrent links behind the authenticated and authorized portal via simple email/password accounts granted by the system administrator. New users may request access via the portal homepage, though a portal administrator must approve new accounts before those users are allowed to log in. Additionally, a portal administrator or publisher must grant each user access to any number of available feeds, as appropriate.

As an additional layer of security, we encourage the use of password-protected compression such as Zip or Gzip prior to publication of sensitive data, though this may limit future tasks related to distributed computing. Nevertheless, payload encryption is encouraged to mitigate the impact of users forwarding their .torrent files via email or other means. Unlike normal BitTorrent trackers and portals, each downloaded .torrent file contains an authentication token for the logged in user. Thus, any such out-of-band forwarding of the .torrent files will include those credentials. While the same forwarding problem exists with sensitive information included in any downloaded .PDF, .DOC, or other file, it is important for users to understand that the .torrent files provided by the portal are *only* for that user, and should not be shared.

Compliance of different systems in the network is, to some extent, given to client sites to enforce according to local policy, as BitTorrent is generally friendly to heterogeneous networks of individual sites. BitTorious (and underlying BitTorrent protocols) are agnostic to computing environment and can be run in any cloud computing environment that provides IaaS, but cannot programmatically determine which data, if any, are subject to special local compliance rules. For uses subject to environmental restrictions such as HIPAA, the BitTorious portal as well as transfer nodes can be hosted on appropriately protected IT infrastructure. Users should consider the nature of data and metadata needing publication prior to deploying an instance of the BitTorious portal.

Most bioinformatics studies involve data bound by some form of “controlled data” access agreement, and data is rarely truly placed in the public domain. When .torrent metadata files are uploaded to BitTorious by a publisher, BitTorious *forces* the enablement of a “private” flag within the torrent as defined by the BEP-0027 draft specification [5], as well as customizes the tracker URL with an authentication token specific to each user joining the swarm. This logical data partitioning enforcement is necessary to address heterogeneous international contexts of licensing rights and statutory restrictions. In the future, ‘omics data may also require treatment as Personal Health Information (PHI), further underscoring the need to proactively develop such tools.

## Results

To test the BitTorious portal software, we established 8 clients at differing hosting environments each running the uTorrent client, which supports the specific configuration and extensions required of BitTorious. Each was configured to force P2P encryption. Each site was physically distinct using various Internet service providers. Sites included Amazon Web Services and Linode platforms, private data centers, as well as home office machinery. Two clients were run on Ubuntu Linux 13.10, three on OSX 10.9 and three on Windows 8.x.

Seven of the accounts were given “subscriber” access to a test RSS feed, with the remaining client granted “publisher” access. Torrents of content sizes ranging 1MiB -10GiB were created with random binary data, as well as 6 reference RNA and several DNA data sets with payloads up to approximately 500GiB. The torrent files were generated with the ctorrent v3.3.2 software using a 4MiB piece size. All operating systems supported the x64 instruction set to avoid potential 32-bit boundary issues. Node-specific settings and network configuration were left to the local operator.

Initial seeding and additional replication of these data performed as expected due to the compliance with the BitTorrent protocol. More importantly, the peer tracker integrated into BitTorious performed well in both the standards-compliant tracker as well as the integrated account management, providing near real-time status updates to the operators via the portal UI, including the percentage completion status and approximate geographic location of each node.

## Discussion

As it is implausible to separate the discussion of technologies from the dollars that fund them, we also note that a distributed approach to data syndication is likely more resilient to disruptive budgetary and political concerns. At sufficient scale, events such as localized national disasters or congressional budget cuts cannot single-handedly end a collaborative effort. At envisioned scale consisting of millions of clients, it would likely take a cataclysmic world-wide physical disruption of both Internet communications infrastructure as well as the destruction of multiple data centers to cause irrecoverable data loss.

Regardless of scale, both BitTorious and BitTorrent are agnostic to payload. Use of the system has been designed with bioinformatics in mind, but any “big data” field with distributed collaboration workflows may make use of their own portal deployment without modification. Functions specific to bioinformatics pipelines have been left to the client side to ensure that the system is truly as generic and repurposable as possible.

## Conclusions

The experiments in this paper were driven by a small number of clients only due to limited availability of suitable computing environments. We intend to focus future research and development on expanding the number of test clients at least several orders of magnitude, portal indexding and search features, as well as a custom client providing anonymous users the ability to donate local storage for any given feed up to a user-specified limit.

While in this study we have focused on demonstrating the feasibility of privatized BitTorrent for genomics studies, the available worldwide goodwill of individual citizen scientists and their unused storage capacity and bandwidth is not to be discounted. World Community Grid (WCG) alone reports around 2,883,000+ active devices on their network. [6] While volunteer *storage* [7,8] has already been suggested, implementations designed based on this model have not surfaced. The natural next step is to develop a lightweight, cross-platform “BitTorious Home” application for anonymous micro-philanthropic volunteerism of local storage. Storing a lower approximate 40 PB of genomic data for archival purposes -- a significant number as described in The Million Cancer Genome Warehouse [9]– at a participation level of 50 % of the devices on a network such as WCG would require approximately 32GiB of donated storage per device for a single replicated data set.

Given both the economic and technological advantages of distributed big data collaboration and archival, solutions such as BitTorious are a compelling proposition worthy of future research and development.

## Availability and requirements

The BitTorious portal software may be downloaded and deployed via the public source code repository at https://github.com/preston/bittorious. Non-administrative evaluation may be performed by requesting an account from the demo site, below, in conjunction with the free uTorrent client software.

- **Project name:** BitTorious
- **Project home page:**
  - ○ Source code: https://github.com/preston/bittorious
  - ○ Demo: https://try.bittorious.com
  - ○ Tutorial: https://try.bittorious.com/getting_started
- **Operating system(s):** Both portal and clients are platform independent.
- **Programming language:** Ruby on Rails, jQuery, Bootstrap
- **Other requirements:** Ruby 2.1 or higher
- **License:** MIT
- **Any restrictions to use by non-academics:** None
